# MPT0B098, a Microtubule Inhibitor, Suppresses JAK2/STAT3 Signaling Pathway through Modulation of SOCS3 Stability in Oral Squamous Cell Carcinoma

**DOI:** 10.1371/journal.pone.0158440

**Published:** 2016-07-01

**Authors:** Hsuan-Yu Peng, Yun-Ching Cheng, Yuan-Ming Hsu, Guan-Hsun Wu, Ching-Chuan Kuo, Jing-Ping Liou, Jang-Yang Chang, Shiow-Lian Catherine Jin, Shine-Gwo Shiah

**Affiliations:** 1 National Institute of Cancer Research, National Health Research Institutes, Miaoli, Taiwan; 2 Department of Life Sciences, National Central University, Taoyuan, Taiwan; 3 Department of Medical Research, Show Chwan Memorial Hospital, Changhua, Taiwan; 4 Institute of Biotechnology and Pharmaceutical Research, National Health Research Institutes, Miaoli, Taiwan; 5 School of Pharmacy, College of Pharmacy, Taipei Medical University, Taipei, Taiwan; 6 Division of Hematology and Oncology, Department of Internal Medicine, National Cheng Kung University Hospital, College of Medical, National Cheng Kung University, Tainan, Taiwan; Seoul National University, REPUBLIC OF KOREA

## Abstract

Microtubule inhibitors have been shown to inhibit Janus kinase 2/signal transducer and activator of transcription 3 (JAK2/STAT3) signal transduction pathway in various cancer cells. However, little is known of the mechanism by which the microtubule inhibitors inhibit STAT3 activity. In the present study, we examined the effect of a novel small-molecule microtubule inhibitor, MPT0B098, on STAT3 signaling in oral squamous cell carcinoma (OSCC). Treatment of various OSCC cells with MPT0B098 induced growth inhibition, cell cycle arrest and apoptosis, as well as increased the protein level of SOCS3. The accumulation of SOCS3 protein enhanced its binding to JAK2 and TYK2 which facilitated the ubiquitination and degradation of JAK2 and TYK2, resulting in a loss of STAT3 activity. The inhibition of STAT3 activity led to sensitization of OSCC cells to MPT0B098 cytotoxicity, indicating that STAT3 is a key mediator of drug resistance in oral carcinogenesis. Moreover, the combination of MPT0B098 with the clinical drug cisplatin or 5-FU significantly augmented growth inhibition and apoptosis in OSCC cells. Taken together, our results provide a novel mechanism for the action of MPT0B098 in which the JAK2/STAT3 signaling pathway is suppressed through the modulation of SOCS3 protein level. The findings also provide a promising combinational therapy of MPT0B098 for OSCC.

## Introduction

The Janus kinase/signal transducer and activator of transcription (JAK/STAT) signal transduction pathway is frequently dysregulated in various human cancer cells [1] and plays a critical role in oncogenesis including proliferation, apoptosis, drug resistance, migration, invasion and angiogenesis [2]. The STAT family member STAT3 has been reported to possess oncogenic potential as constitutive activation in oral squamous cell carcinoma (OSCC) and transduce signals elicited by various cytokines leading to regulation of specific target genes that contribute to a malignant phenotype [3–5]. Furthermore, targeting STAT3 with dominant negative mutants of STAT3 or antisense oligonucleotides specific for the STAT3 DNA sequence causes reversion of the malignant phenotype of squamous cell carcinoma [6, 7], suggesting that STAT3 is a key mediator for the pathogenesis of these cancers. There are two classical negative feedback regulators for the JAK/STAT signaling pathway, the protein inhibitors of activated STATs (PIAS) and the suppressors of cytokine signaling (SOCS), through which the STAT pathway is silenced by masking STAT binding sites on the receptors, by binding to JAKs to inhibit their kinase activity, or by targeting proteins for proteasomal degradation through ubiquitination [8, 9]. Among these negative regulators, SOCS3 is known to attenuate interleukin-6 (IL-6) induced STAT3 activation [10, 11]. An *in vivo* study has shown that *Socs3*-deficient mice produced a prolonged activation of STAT3 after IL-6 treatment [10], indicating a crucial role of SOCS3 in IL-6/JAK/STAT signaling axis. Moreover, loss of SOCS3 expression has been described in head and neck squamous cell carcinoma (HNSCC) [12]. Experimental overexpression of SOCS protein in cancer cells results in growth suppression and apoptosis induction [12], strongly suggesting that SOCS proteins may function as tumor suppressors. Thus, SOCS3 is regarded as a useful diagnostic molecule and a potential therapeutic target for HNSCC.

To date, more than 90% of HNSCC belongs to OSCC in the South-East Asia, including Taiwan [13]. Despite the fact that most patients who are readily amenable to clinical examination and diagnosed at an early stage have an excellent survival rate, the 5-year survival rate for those patients with loco-regional recurrences and neck lymph metastasis has not significantly improved over the past years [14]. Thus, there is a need for a better understanding of the biological nature of oral cancers in order to develop novel strategies to improve the efficacy of the treatment. At present, the usage of chemotherapy drugs available for oral cancers, such as 5-fluorouracil (5-FU) and cisplatin, is limited due to their side effects, drug resistance and non-specificity [15, 16]. As a result, more attention has been drawn to the combinational approach aiming to improve the efficacy of the chemotherapeutic drugs on OSCC tumorigenesis and progression [17–19].

In the present study, we used a novel small-molecule microtubule inhibitor, 7-aryl-indoline-1-benzene-sulfonamide (MPT0B098) [20], to examine whether a microtubule-based chemotherapy modulates the JAK2/STAT3/SOCS3 signal pathway. We found that MPT0B098 could delay the turnover of SOCS3 protein in OSCC cell lines and resulted in JAK2/STAT3 inactivation and induction of apoptosis. Inhibition of endogenous SOCS3 significantly reduced the MPT0B098-induced apoptosis in oral cancer cells, whereas overexpression of SOCS3 induced the apoptosis. Furthermore, treatment with MPT0B098 in combination with cisplatin or 5-FU caused significantly apoptosis as compared to the treatment with a single agent or the combination of cisplatin and 5-FU. Taken together, our results uncover a novel mechanism for the action of the microtubule inhibitor MPT0B098 on SOCS3 modulation in OSCC cells. They also provide a promising therapeutic strategy for OSCC.

## Materials and Methods

### Cell culture and chemicals

Human oral keratinocytes (HOK) were purchased from ScienCell (Carlsbad, CA, USA) and cultured according to the manufacturer’s instructions. The culture conditions for all the OSCC cell lines are summarized as previously described [21]. All cells were cultured at 37°C in a 5% CO_2_ atmosphere and maintained in the presence of 10% fetal bovine serum (FBS, Kibbutz BeitHaemek, Israel) for approximately 3 months after resuscitation from the frozen aliquots. The cells used for the experiments were lower than 20 passages. All culture media were purchased from Invitrogen. Sodium pyruvate was obtained from Merck and hydrocortisone from Sigma-Aldirch. The chemical synthetic procedure of MPT0B098 was described previously [22].

### Protein extraction and Western blot analysis

The OSCC cells were lysed in a lysis buffer containing 50 mM Tris-HCl, 1% NP-40, 150 mM NaCl, 0.1% SDS, 1 mM PMSF, 1mM Na_3_VO_4_ and protease inhibitor cocktail (1:1000) (Sigma-Aldrich, Inc.). Protein concentrations then were determined by the BCA assay kit (Thermo, USA) according to the manufacturer’s instructions. Equal amount of protein lysates were loaded onto 10~12% SDS polyacrylamide gels and subjected to electrophoresis, followed by transferring the proteins to poly-vinylidene fluoride membrane (Pall Life Sciences, Glen Cove, NY). The membranes then were probed with antibodies specific for Bcl-2 (sc-509) (Santa Cruz, Heidelberg, Germany), phosphor-JAK2 (Millipore, MA, USA), JAK1 (#E021119), phosphor-JAK1 (#E021149) (EnoGene, NY, USA), Mcl-1 (#GTX102026) (GeneTex, Inc, Irvine, CA, USA), Survivin (#2463), Pim-1 (#2409), JAK2 (#2863), phosphor-STAT3 (#2236) (Epitomics Inc., Burlingame, CA), STAT3 (#610189) (BD Biosciences, NJ, USA), α-tubulin (#MS-581-P) (Thermo, CA, USA), caspases-3 (#9662), PARP (#5625), TYK2 (#9312), phosphor-TYK2 (#9321), SOCS1 (#3950), SOCS2 (#2779), SOCS3 (#2932), PIAS1 (#3550), and PIAS3 (#4164) (Cell signaling, USA). The anti-GAPDH antibody (Thermo, CA, USA) was used as an internal control. Signals from HRP-coupled secondary antibodies were visualized by the enhanced chemiluminescence (ECL) detection system (PerkinElmer, Waltham, MA) and the chemiluminescence was exposed onto Kodak X-Omat film (Kodak, Chalon/Paris, France).

### RNA extraction and reverse transcription-polymerase chain reaction (RT-PCR) assay

Total RNA was isolated from OSCC cells with the TRIzol reagent (Life Technologies, Gaithersburg, MD) according to the manufacturer’s instructions. First strand cDNA was synthesized using random hexamer primers and SuperScript III reverse transcriptase (Invitrogen, Carlsbad, CA). PCR was run on a Biometra T3000 thermocycler (Biometra GmbH, Germany). The reaction cycles included an initial denaturation step at 95°C for 5 min, followed by 30 cycles of denaturation at 95°C for 30 s, annealing at 60°C for 30 s, extension at 72°C for 30 s and one cycle of final extension for 10 min at 72°C. The housekeeping gene glyceraldehyde 3-phosphate dehydrogenase (GAPDH) was used as an internal control. The sequences of the primers used for PCR were as follows: SOCS3 5’-TATG CGGC CAGC AAAG AATC A-3’ (forward), 5’-CGGG CAAT CTCC ATTG GCT-3’ (reverse); GAPDH 5’-GAA GGT GAA GGT CGG AGT-3’ (forward), 5’-GAA GAT GGT GAT GGG ATT TC -3’ (reverse). PCR products were subjected to electrophoresis on 2% agarose gel and visualized on UVP GDS-8000 Bioimaging System (UVP, CA, USA) with 0.01% of SYBR® Safe (Invitrogen, Carlsbad, CA, USA) staining.

### Plasmids and transfection

To construct the human SOCS3 expression vector, a 677-base pair fragment of SOCS3 was amplified by PCR using the SOCS3 cloning primers: 5’-GGGG ATCC GCCA CCAT GGTC ACCC ACAG CAAG-3’ (forward) and 5’-GGGA ATTC TTAA GCGG GGCA TCGT AC-3’ (reverse), and then cloned into the BamHI/EcoRI sites of pcDNA3.1^+^ vector (Invitrogen, Gaithersburg, MD). For gene knockdown experiments, the shRNA clones for SOCS3, STAT3 and their control pLKO_TRC vector (NS) were obtained from the National RNAi Core Facility (Academia Sinica, Taiwan). The plasmids were transiently transfected into OSCC cells using Lipofectamine 2000 (Invitrogen, CA, USA) according to the manufacturer’s protocol. Briefly, Lipofectamine and plasmid were dissolved in Opti-MEM® I Reduced Serum Medium (Gibco, NY, USA) separately and incubated at room temperature for 5 min. The two reagents then were mixed and incubated for additional 20 min at room temperature. Subsequently, the Lipofectamine-plasmid mixture was added drop-wise onto the OSCC cells grown in the culture medium containing 10% FCS After incubation for 48 hrs, the cells were harvestedfor further analysis.

### Preparation of monomer and polymer fractions of microtubule

The monomer and polymer fractions of microtubule were prepared following the previously described methods with slight modifications [23]. Briefly, cells were seeded in 60-mm plastic petri dishes and treated with testing drugs for indicated times. Cells then were washed twice with cold phosphate-buffer saline (PBS), followed by extraction with microtubule-stabilizing buffer (MSB) (85 mM PIPES, pH 6.93, 1 mM EGTA, 1 mM MgCl_2_, 2 M Glycerol, and Sigma protease inhibitor cocktail) containing 0.5% Triton X-100. After 3 min, the Triton extract (monomer fraction) was gently removed from the dish and transferred to an eppendrof tube, and 1/5 volume of 5x SDS sample buffer (10% SDS, 325 mM Tris-HCl, pH 6.8, 30% glycerol, 250 mM DTT and 1 mM phenylmethylsulfonyl fluoride) was added into the tube. The remaining part (polymer fraction) in the petri dish was gently washed twice with cold MSB. The polymer fraction then was extracted with MSB and mixed into 1/5 volume of 5x SDS sample buffer. Both extracts were boiled for 10 min and stored at -80°C till western blot analysis.

### Cell viability assay

The OSCC cells were harvested in the exponential growing phase and seeded into 96-well plates (3000 cells/well). After overnight incubation, 200 μl of culture medium containing the test compound was dispensed into each well. Following 72-h treatment, 3-[4,5-dimethylthiazol-2-yl]-2,5-diphenyl tetrazolium bromide (MTT) dye (Calbiochem, CA, USA) was added. After 2 hrs of incubation, the medium and MTT dye were removed by slow aspiration and 100μl of dimethyl sulfoxide (DMSO) was added to dissolve the remaining MTT formazan crystals. The absorbance at 550 nm was measured using a 96-well plate SpectraMax 250 reader (Molecular Devices, CA, USA).

### Cell cycle analysis

The OSCC cells were seeded in 12-well plates (2x10^5^ cells/well) and treated with drugs for 24 hrs. Cells then were trypsinized, washed with PBS, and fixed in 70% ethanol at -20°C. After fixation, cells were washed twice with PBS and incubated in 0.5% Triton X-100/PBS containing 1 mg/ml RNase A at 37°C for 30 min. Subsequently, the cells were stained with propidium iodide (PI) at the final concentration of 30 μg/ml. Samples were measured using the FACscan flow cytometer (Becton Dickinson).

### Apoptosis assay

Caspase-3 protease activity was measured by the Caspase-3/CPP32 activity Colorimetric Assay kit (Biovision Incorporate, CA, USA). In brief, cells were seeded in 6-well plates (2x10^6^ cells/well) and treated with drugs for 24 hrs. The cells then were harvested and centrifuged at 4°C for 5 min. The cytosolic extracts were prepared by repeated cycles of freezing and thawing of the cells in 50 μl of lysis buffer. The lysates were centrifuged at 10,000 xg for 5 min. Protein concentrations were determined by the BCA assay kit (Thermo, USA) according to the manufacturer’s instructions. Cell lysates (100 μg) were then diluted with 50μl of lysis buffer followed by addition of 50 μl of the 2X reaction buffer. After incubation in the presence of the fluorescence substrate Ac-DEVD-pNA (200 μM) at 37°C for 2 hrs in dark, the absorbance at 405 nm was determined using a SpectraMax 250 reader (Molecular Devices, CA, USA). For the Annexin V-fluorescein isothiocyanate (FITC)/PI assay, testing cells were washed with cold PBS twice, and then resuspended in the buffer containing Annexin V-FITC and PI. The mixture was kept in dark for 15 min at room temperature before analysis by flow cytometry.

### Immunofluorescent staining and microscopy

For immunofluorescence microscopy, the cells were fixed with freshly prepared 3% paraformaldehyde (Merck, Whitehouse Station, NJ) for 10 min, permeabilized with 0.1% Triton-X/PBS for 10 min, followed by blocking with 3% bovine serum albumin (BSA) in PBS at 37°C for 1 hr. The cells then were incubated with a mouse α-tubulin monoclonal antibody (MS-581-P, Thermo; diluted in 3% BSA/PBS) for 1 hr, followed by incubation with a FITC-conjugated secondary antibody (Santa Cruze) at room temperature for 1 hr in dark. Finally, the nuclei were counterstained with 4′, 6-diamidino-2-phenylindole (DAPI) at room temperature for 5 min. The coverslips were mounted with ProLong Gold anti-fade reagent (Invitrogen, Carlsbad, CA). The images were captured using the Leica TCS SP5 Confocal Microscopy Workstation (Leica, Wetzlar, Germany).

### Statistical analysis

The data were expressed as the mean ± standard error (SE) from at least three independent experiments. Differences between various treatment groups were assessed by ANOVA and Student's t-test. A *p*-value < 0.05 was considered as significant. Calculations were performed using Graph Pad Prism Ver. 4.01 (San Diego, CA).

## Results

### MPT0B098 inhibits the proliferation and tubulin polymerization in OSCC cells

**Fig 1:**
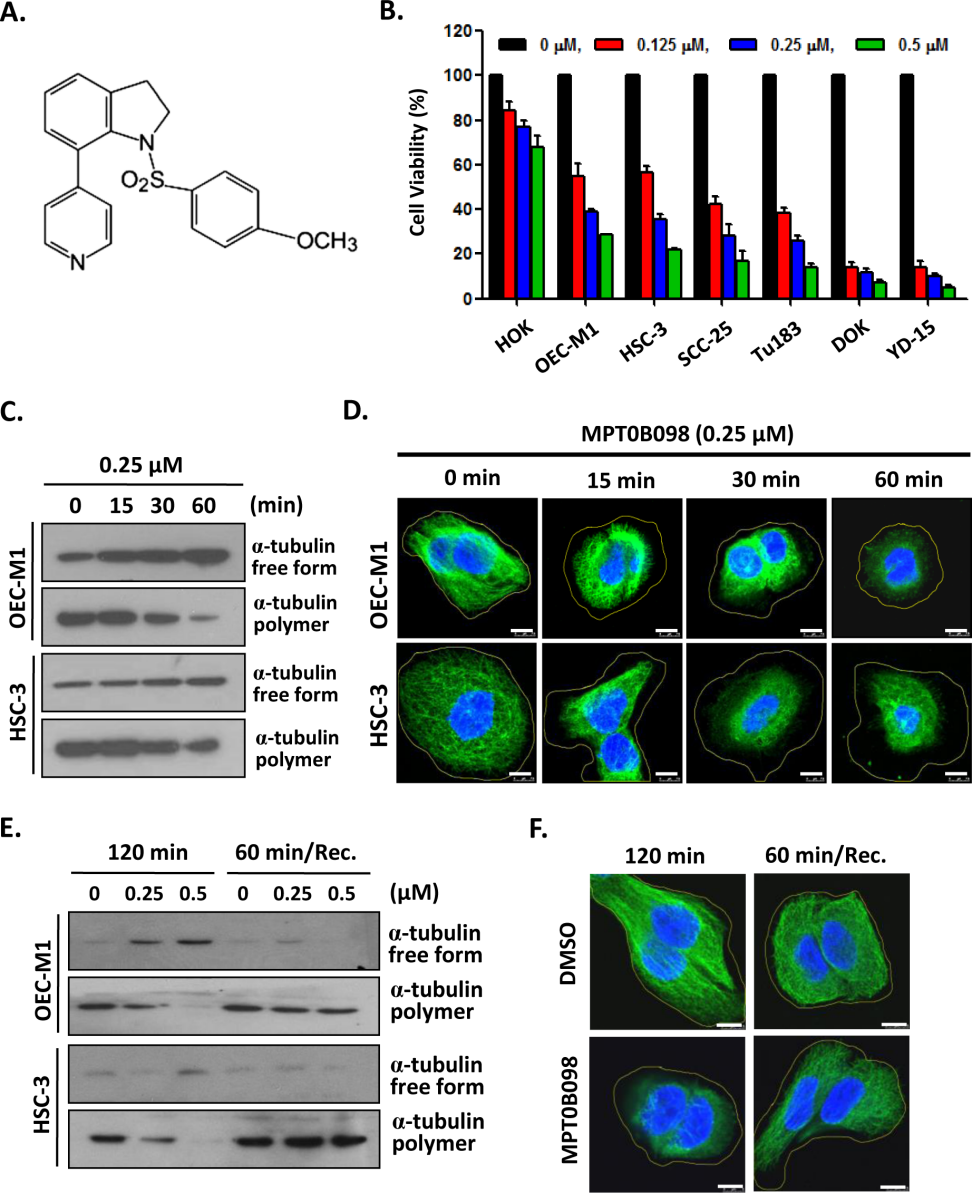
MPT0B098 inhibits the proliferation and induces microtubules depolymerization in OSCC cells. (**A**) Chemical structure of MPT0B098. (**B**) OSCC cells were treated with increasing concentrations of MPT0B098 for 72 hrs and the cell viability was assessed by MTT assay. Data are presents as mean ± SE relative to DMSO vehicle control (indicated as 0 μM) from three replicate experiments. *, *p*<0.05; **, *p*<0.01; ***, *p*<0.001. (**C**) OEC-M1 and HSC-3cells were incubated at 37°C from 0~60 min in the presence of 0.25 μM of MPT0B098. Free form and polymer form of microtubule were purified and assessed by Western blot analysis. (**D**) OEC-M1 and HSC-3 cells were treated with 0.25 μM of MPT0B098 from 0~60 min. Cells were fixed and then immunostained with anti-α-tubulin (green) antibody and then stained with DAPI (blue), followed by confocal microscopy. Scale bar = 7.5 μm. (**E**) OEC-M1 and HSC-3 cells were treated with MPT0B098 for 120 min at 37°C. For recovery (Rec.) assay, cells were treated with MPT0B098 for 60 min and then the drug was washed out to allow the microtubules to repolymerize for another 60 min. Cell lysates were analyzed by western blot using the anti-α-tubulin antibody. (**F**) The recovery assay was also done in OEC-M1 cells for immunostaining. After drug treatment, OEC-M1 cells were fixed and then immunostained with anti-α-tubulin (green) antibody and then stained with DAPI (blue), followed by confocal microscopy. Scale bar = 7.5 μm.

In an initial study, the effect of MPT0B098 (Fig 1A) on cell viability was assessed in various human OSCC cells using the MTT assay. As shown in Fig 1B, MPT0B098 inhibited cell growth of all OSCC cell lines tested in a dose-dependent manner, and the IC_50_ concentrations for these cell lines, including OEC-M1, HSC-3 SCC-25, Tu183, DOK and YD-15, were ranging from 0.14 to 0.45 μmol/L (Table 1).

**Table 1:** IC_50_ values of MPT0B098 on cell viability in HOK and OSCC cell lines.

Cells	Origin	IC_50_ (μmole/L)
HOK	Oral keratinocyte	6.30±0.30
OEC-M1	Gingival squamous cell carcinoma	0.45±0.01
SCC-25	Tongue squamous cell carcinoma	0.36±0.02
Tu183	Tongue squamous cell carcinoma	0.31±0.02
HSC-3	Tongue squamous cell carcinoma	030±0.04
DOK	Oral epithelial dysplasia	0.14±0.05
YD-15	Tongue mucoepidermoid carcinoma	0.14±0.13

On the other hand, the normal human oral keratinocytes (HOK) exhibited less susceptibility to the inhibitory effect of MPT0B098 with the IC_50_ of 6.3 μmol/L (Fig 1B and Table 1). The differential toxicity of MPT0B098 on normal keratinocytes and oral cancer cells indicates a potential of the compound for clinical use. We further examined whether tubulin depolymerization was induced by MPT0B098 in the OSCC cells. For this purpose, we isolated soluble non-polymerized tubulin and insoluble polymerized tubulin from OEC-M1 and HSC-3 cells after MPT0B098 treatment. The tubulin polymerization assay showed that MPT0B098 inhibited tubulin polymerization in a time- and concentration-dependent manner (Fig 1C and S1 Fig). The effect of MPT0B098 on microtubule structure then was determined by immuno-fluorescence microscopy using anti-α-tubulin antibody. The results showed that MPT0B098 disrupted microtubule network in a time-dependent manner similar to that of tubulin polymerization (Fig 1D). Furthermore, to test the capacity of MPT0B098 on depolymerization of the tubulin network, we treated the cancer cells with the compound and then measured the recovery of the tubulin network after MPT0B098 washout. We found that the recovery of microtubules network, as shown by the dense tubulin staining and longer microtubules fibers, could be observed in OSCC cells at 60 min after MPT0B098 washout (Fig 1E and 1F).

### MPT0B098 induces cell cycle arrest and apoptosis in OSCC cells

**Fig 2:**
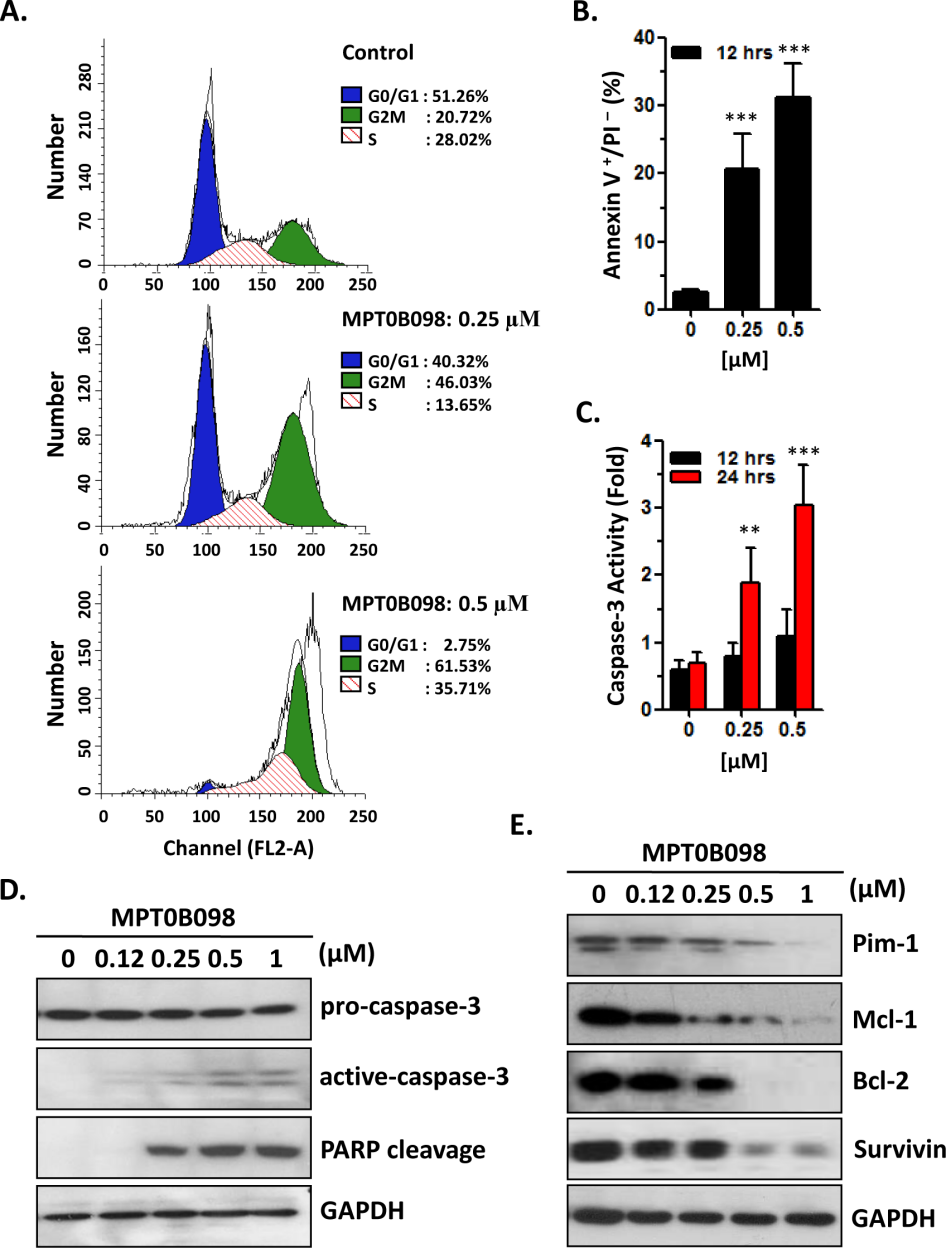
MPT0B098 induces the cell cycle arrest and apoptosis. (**A**) OEC-M1 cells were treated with 0.25 or 0.5 μM of MPT0B098 for 12 hrs. Cells were then evaluated for effects on cell cycle using PI staining and analyzed by flow cytometry. Percentages of cells in different phases were shown. The data are representative of three independent experiments. (**B**) OEC-M1 cells were treated with different concentrations of MPT0B098 for 12 hrs. Apoptosis was assessed by annexin V/PI staining and analyzed by flow cytometry. The data are represented as mean ± SE; ***, *p*<0.001 versus vehicle control. (**C**) OEC-M1 cells were incubated with various concentrations of MPT0B098 for 12~24 hours and caspases-3 activity was assessed. The data are represented as mean ± SE; **, *p*<0.01; ***, *p*<0.001 versus vehicle control. (**D**) OEC-M1 cells were treated with different concentrations of MPT0B098 for 24 hrs. The proteolytic cleavage of caspase-3 and PARP were determined by Western blot analysis. GAPDH was used as protein loading control. (**E**) OEC-M1 cells were treated with different concentrations of MPT0B098 for 24 hrs. Effects on the expression of Pim-1, Mcl-1, Bcl-2 and survivin were determined by western blot analysis. GAPDH was used as protein loading control.

To determine the effects of MPT0B098 on cell cycle and apoptosis, OEC-M1 cells were treated with different concentrations of MPT0B098 and the cell cycle progression and the alteration of apoptotic markers in the cells were analyzed. As shown in Fig 2A, MPT0B098 induced a dose-dependent G_2_/M arrest at 12 h following the drug exposure. At the drug concentrations of 0.25 and 0.5 μmol/L, the population of OEC-M1 cells in G_2_/M phase was 46.03% and 61.53%, respectively (Fig 2A). In addition, exposure of MPT0B098 for 12 h also caused a dose-dependent induction of cell apoptosis as determined by annexin V-FITC staining but not PI staining (Annexin V^+^/PI^-^) (Fig 2B). Notably, dose-dependent increases in caspase-3 activation and PARP cleavage were also observed in MPT0B098-treated cells (Fig 2C and 2D). Moreover, the effect of MPT0B098 on the levels of anti-apoptotic proteins in OSCC cells was also investigated. As shown in Fig 2E, MPT0B098 caused a dose-dependent down-regulation of Bcl-2, Pim-1, Survivin, and Mcl-1 proteins. These findings suggest that in OSCC cells the induction of apoptosis by MPT0B098 may be mediated by its inhibition on the expression of these anti-apoptotic proteins.

### MPT0B098 down-regulates the protein level and activity of JAK2, TYK2 and STAT3 protein

**Fig 3:**
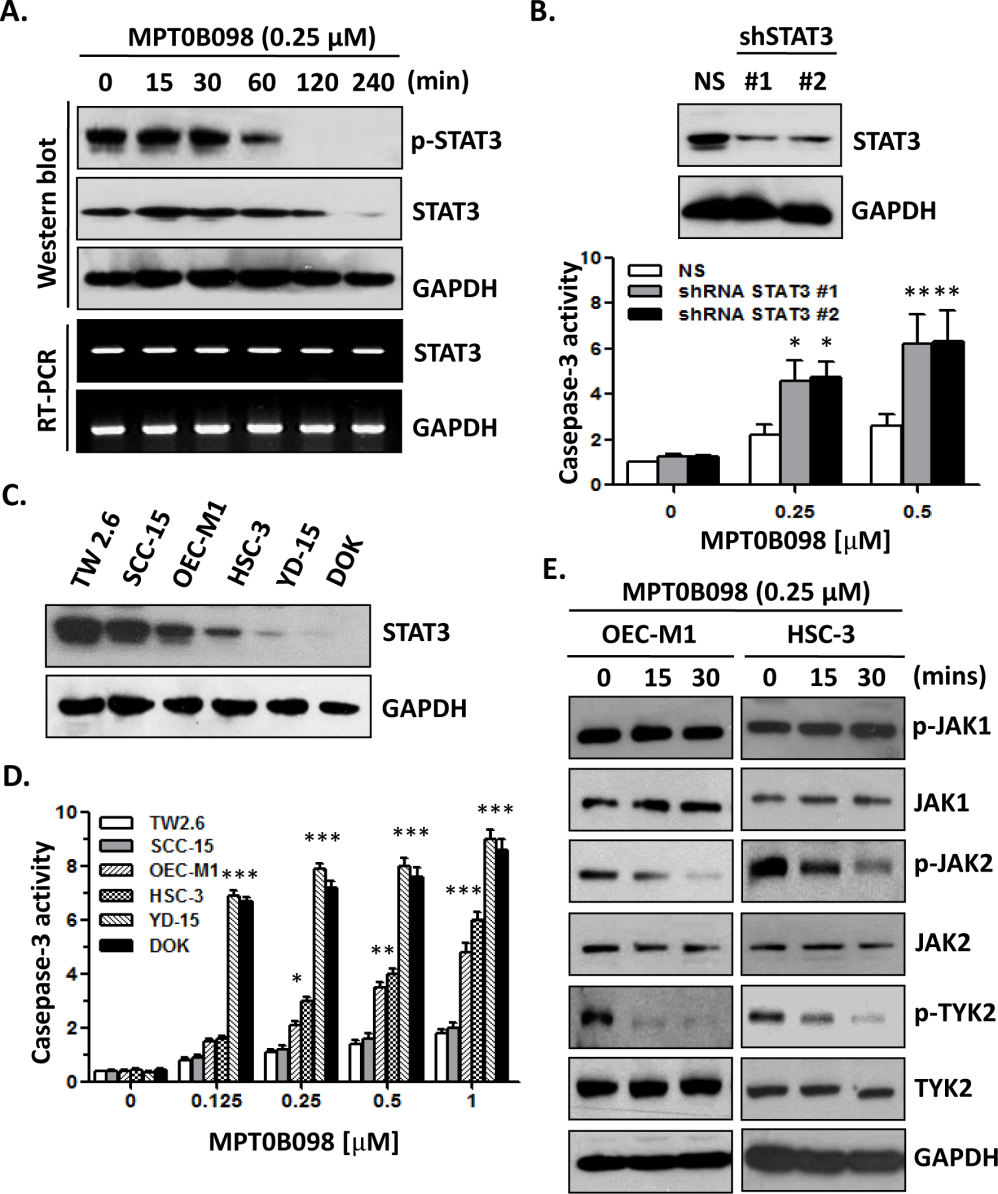
MPT0B098 modulates JAK2/STAT3 pathway in OSCC cells. (**A**) OEC-M1 cells were treated with MPT0B098 (0.25 μM) for the indicated times. The phosphorylated STAT3 (pSTAT3) and total level of STAT3 were determined by Western blotting. The level of STAT3 mRNA was determined by RT-PCR. GAPDH was used as loading control. (**B**) Western blot analysis of STAT3 levels after 48 hrs transfection of OEC-M1 cells with control shRNA (NS) or two shRNA constructs (shSTAT3 #1, #2) against STAT3. GAPDH was used as loading control. These shRNA transfactants were treated MPT0B098 for 24 hrs and caspases-3 activity was assessed. The data are represented as mean ± SE; *, *p*<0.05; **, *p*<0.01 versus vehicle control. (**C**) Western blot analysis of endogenous STAT3 protein level in OSCC cells. GAPDH was used as loading control. (**D**) OSCC cells were incubated with various concentrations of MPT0B098 for 24 hrs and caspases-3 activity was assessed. Data are presents as mean ± SE relative to vehicle control from three replicate experiments. *, *p*<0.05; **, *p*<0.01; ***, *p*<0.001. (**E**) OEC-M1 and HSC-3 cells were treated with 0.25 μM of MPT0B098 for indicated times. The phosphorylated proteins (p-JAK1, p-JAK2 and p-TYK2) and total level of proteins (JAK1, JAK2 and TYK2) were determined by Western blotting. GAPDH was used as loading control.

Recent evidence indicates that the transcription factor STAT3 may act as an oncogene by activating the expression of several anti-apoptotic genes, such as Bcl-2, Pim-1, Survivin, and Mcl-1 [3]. To determine whether STAT3 is involved in the apoptotic effect of MPT0B098on cancer cells, we examined STAT3 activation and expression in MPT0B098 treated OSCC cell lines. The results showed that the STAT3 phosphorylation and STST3 protein level were markedly decreased at 1 h and 2 h, respectively, after MPT0B098 treatment, while the level of STAT3 mRNA was not changed (Fig 3A). Using the shRNA knockdown approach to down-regulate the STAT3 protein level, we found that the MPT0B098-induced apoptosis in OEC-M1 cells was significantly enhanced (Fig 3B). Furthermore, MPT0B098-induced apoptosis was inversely correlated with the endogenous STAT3 protein levels (Fig 3C and 3D), indicating that STAT3 activity is involved in the chemoresistance of OSCC. Because the JAK/STAT3 pathway is the canonical signaling pathway of STAT3 activation, we further investigated whether the JAK activity is affected by MPT0B098. Similar to the effect on STAT3 inhibition, MPT0B098 also showed an inhibition of JAK2 and TYK2 tyrosine phosphorylation in OEC-M1 and HSC-3 cells, but it did not affect JAK1 expression or phosphorylation (Fig 3E). Taken together, our data suggest that the STAT3 signaling is involved in the MPT0B098-induced apoptosis in OSCC cells.

### MPT0B098 down-regulates JAK2, TYK2 and STAT3 through SOCS3 negative feedback modulation

**Fig 4:**
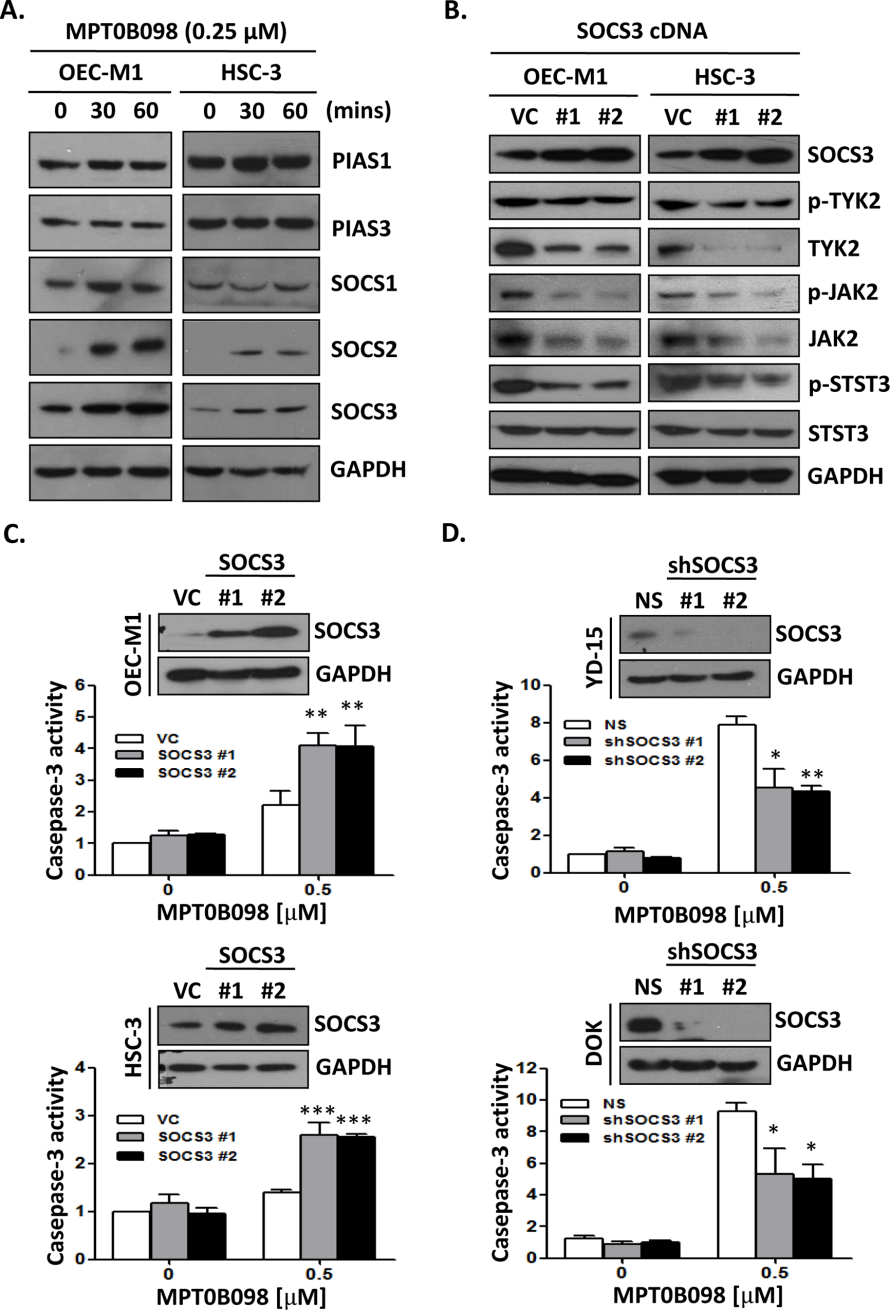
MPT0B098 modulates JAK2/STAT3 pathway via SOCS3 accumulation. (**A**) OEC-M1 and HSC-3 cells were treated with 0.25 μM of MPT0B098 for indicated times. The protein level of PIAS1, PIAS3, SOCS1, SOCS2, and SOCS3 were determined by Western blotting. GAPDH was used as loading control. (**B**) OEC-M1 and HSC-3 cells were tranfected with control vector (VC) and construct containing the SOCS3-coding region (SOCS3 cDNA #1, #2) for 48 hrs. The phosphorylated proteins (p-JAK2, p-TYK2 and p-STAT3) and total level of proteins (SOCS3, JAK2, TYK2 and STAT3) were determined by Western blotting. GAPDH was used as loading control. (**C**) Western blot analysis of SOCS3 levels after 48 hrs transfection of OEC-M1 and HSC-3 cells with control vector (VC) or two SOCS3 constructs (SOCS3 #1, #2). GAPDH was used as loading control. These SOCS3 overexpression transfactants were treated MPT0B098 for 24 hrs and caspases-3 activity was assessed. The data are represented as mean ± SE; **, *p*<0.01 versus vehicle control. (**D**) Western blot analysis of SOCS3 levels after 48 hrs transfection of YD-15 and DOK cells with control shRNA (NS) or two shSOCS3 constructs (shSOCS3 #1, #2). GAPDH was used as loading control. These shRNA transfactants were treated MPT0B098 for 24 hrs and caspases-3 activity was assessed. The data are represented as mean ± SE; ***, *p*<0.001 versus vehicle control.

The STAT signaling can be inhibited by two signal pathways that involve the SOCS family and the PIAS family members. These signal molecules act as negative feedback regulators to prevent further JAK/STAT signal activation [24, 25]. Thus, it was hypothesized that these negative feedback regulators may be involved in the MPT0B098 regulation of the JAK/STAT activity. To address this, the protein levels of the SOCS and PIAS family members in MPT0B098-treated OEC-M1 and HSC-3 cells were measured. The immunoblotting results showed that only SOCS2 and SOCS3 levels were markedly elevated in these cells, whereas the levels of PIAS1, PIAS3 and SOCS1 proteins remained unchanged (Fig 4A). We further explored the effect of SOCS2 and SOCS3 on JAK/STAT signaling pathway in OSCC cells. We found that the total protein and phosphorylation levels of JAK2, TYK2 and STAT3 were significantly decreased in the OSCC cells transfected with a SOCS3 cDNA construct (Fig 4B). However, overexpression of SOCS2 did not change the total protein and phosphorylation levels of JAK2, TYK2 and STAT3 (S2 Fig). We then investigated the role of SOCS3 in MPT0B098-induced apoptosis in OSCC cells. Overexpression of SOCS3 protein in OEC-M1 and HSC-3 cells markedly increased the MPT0B098-induced apoptosis (Fig 4C).However, knockdown of SOCS3 protein in DOK and YD-15 cells significantly attenuated the MPT0B098-induced apoptosis (Fig 4D). These results suggest that the induction of cancer cell apoptosis by MPT0B098 may be mediated by a negative modulation of SOCS3 on JAK2/STAT3 signaling pathway.

### MPT0B098 promotes SOCS3 binding to JAK2 and TYK2

**Fig 5:**
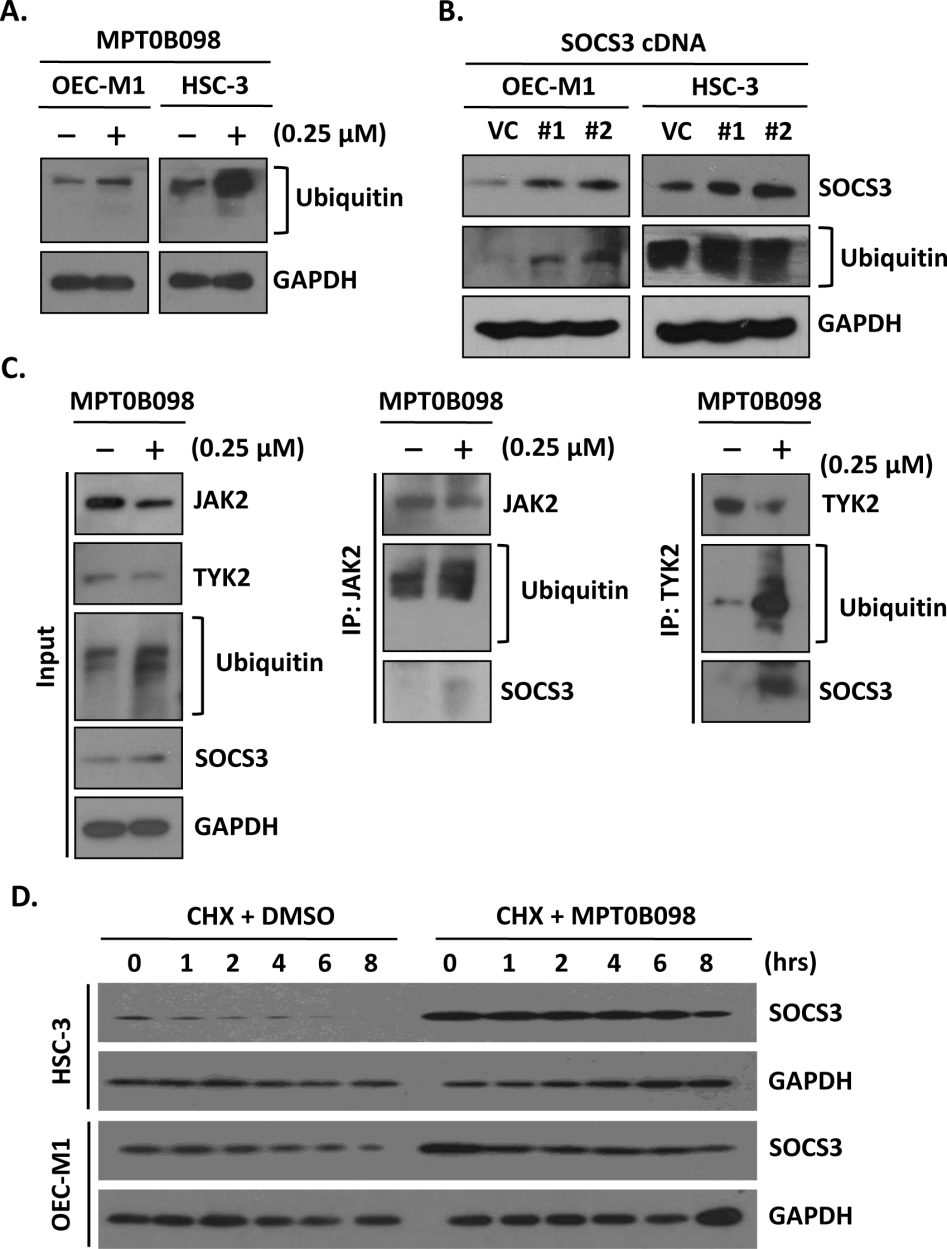
MPT0B098-induced SOCS3 accumulation enhances the ubiquitination of JAK2 and TYK2. (**A**) OEC-M1 and HSC-3 cells were treated with 0.25 μM MPT0B098 for 24 hrs and analyzed by Western blot for protein ubiquitination (ubiquitin). (**B**) Western blot analysis of SOCS3 and ubiquitin levels after 48 hrs transfection of OEC-M1 and HSC-3 cells with control vector (VC) or two SOCS3 constructs (SOCS3 #1, #2). (***C***) OEC-M1 cells were treated with 0.25 μM MPT0B098 for 24 hrs. The whole cell lysates (input) were immunoblotted with antibodies to JAK2, TYK2, ubiquitin and SOCS3 (*left*). Drug treated cell lysates were immunoprecipitated with anti-JAK2 (IP: JAK2) (*middle*) or anti-TYK2 (IP: TYK2) and then western blot for JAK2, TYK2, ubiquitin and SOCS3. (**D**) OEC-M1 and HSC-3 cells were treated with 10 μg/ml of cycloheximide in the presence of a vehicle (DMSO) or 0.25 μM MPT0B098 for the indicated times. The SOCS3 levels were determined by Western blotting. GAPDH was used as protein loading control.

A recent study has indicated that a microtubule-depolymerizing drug, nocodazole, can delay the protein turnover of SOCS1 [26]. This report, together with our observations that MPT0B098 induced SOCS3 protein accumulation and then enhanced the apoptosis of OSCC cells (Fig 4), led us to hypothesize that this microtubule-targeting drug MPT0B098 may trigger an interaction between SOCS3 and tubulin, which then negatively regulates STAT3 activity by inactivating JAK2 and TYK2 via ubiquitination and degradation. To test this hypothesis, we first evaluated the ability of MPT0B098 to induce protein ubiquitination. Western blot analysis revealed that the ubiquitination of cellular proteins was markedly elevated in OEC-M1 and HSC-3 cells treated with MPT0B098 (Fig 5A). This finding was consistent with the result observed in the cells overexpressing SOCS3 protein (Fig 5B). To examine whether the interaction between SOCS3 and JAK2 or TYK2 was affected by MPT0B098, we performed pull-down assay with anti-JAK2 or anti-TYK2 antibodies and Western blot for detection of SOCS3 and ubiquitin in OEC-M1 cells. As shown in Fig 5C, the levels of JAK2- and TYK2-associated SOCS3 were significantly increased following treatment with MPT0B098, and the JAK2 and TYK2 levels were decreased by ubiquitination and degradation. Furthermore, cycloheximide treatment of OEC-M1 and HSC-3 cells in the presence of MPT0B098 showed a dramatic increase in the level of SOCS3 protein compared with the cells treated with the vehicle DMSO, indicating that the turnover rate of SOCS3 can be prolonged by MPT0B098 (Fig 5D). These data suggest that the SOCS3 protein level is affected by intact microtubule structure and the microtubule-depolymerizing drug MPT0B098 can induce the accumulation of SOCS3 protein for further ubiquitination and degradation of its binding partners.

### MPT0B098 in combination with cisplatin or 5-FU significantly induces apoptosis in OSCC cells

**Fig 6:**
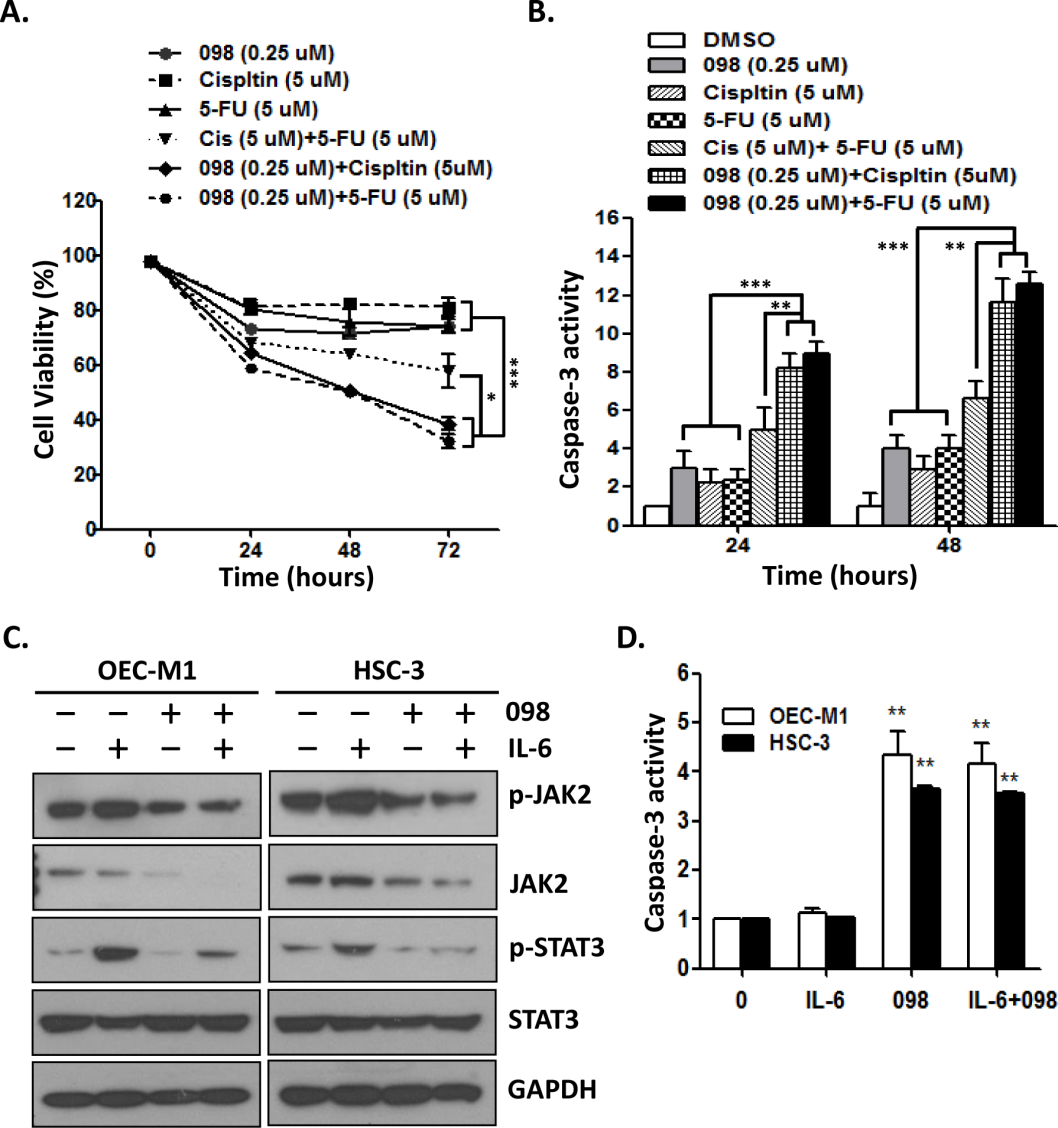
Combination treatment significantly induces apoptosis in OSCC cells. (**A**) OEC-M1 cells were treated with MPT0B098 (0.25 μM), cisplatin (5 μM), 5-fluorouracil (5-FU, 5 μM), cisplatin (5 μM)+5-FU (5 μM), 098 (0.25 μM)+ 5-FU (5 μM) and 098 (0.25 μM)+ cisplatin (5 μM) for the indicated times. The cell viability was assessed by MTT assay. (**B**) OEC-M1 cells were treated with MPT0B098 (0.25 μM), cisplatin (5 μM), 5-fluorouracil (5-FU, 5 μM), cisplatin (5 μM)+5-FU (5 μM), 098 (0.25 μM)+ 5-FU (5 μM) and 098 (0.25 μM)+ cisplatin (5 μM) for the indicated times and the caspase-3 activity was assessed. (**C**) OEC-M1 and HSC-3 cells were stimulated with IL-6 (10 ng/ml) in the presence or absence of MPT0B098 (0.25 μM) for the indicated times. Whole cell lysates were immunoblotted with antibodies to phosphorylated proteins (p-JAK2 and p-STAT3) and total level of proteins (JAK2 and STAT3). GAPDH was used as protein loading control. (**D**) OEC-M1 and HSC-3 cells were stimulated with IL-6 (10 ng/ml) in the presence or absence of MPT0B098 (0.25 μM) for 24 hrs and the caspase-3 activity was assessed. All data are presents as mean ± SE relative to DMSO vehicle control from three replicate experiments. *, *p*<0.05; **, *p*<0.01; ***, *p*<0.001.

We further examined the effect of MPT0B098 alone or in combination with two clinical drugs, cisplatin and 5-FU, on cell survival and apoptosis. Treatment of OEC-M1 cells with MPT0B098 (0.25 μM), cisplatin (5 μM) or 5-FU (5 μM) alone produced 26%, 19% and 26% inhibition, respectively, of cell survival (Fig 6A). The combination treatments, including cisplatin (5 μM) plus 5-FU (5 μM), MPT0B098 (0.25 μM) plus cisplatin (5 μM) or MPT0B098 (0.25 μM) plus 5-FU (5 μM), resulted in greater inhibition of cell survival at 43%, 62% and 68% inhibition, respectively (Fig 6A). Among these combinations, MPT0B098 plus cisplatin or plus 5-FU showed higher inhibition of cell survival as compared to the combination treatment of cisplatin and 5-FU (Fig 6A). Parallel to their inhibitory effect on cell survival, the combination of MPT0B098 with cisplatin or 5-FU produced greater induction of apoptosis in OEC-M1 cells as compared to the drugs treated alone or the combination of cisplatin and 5-FU (Fig 6B). Growing evidence suggests that JAK2/STAT3 signaling can be activated by IL-6 and this activation is required for tumor initiation and progression [27, 28]. To test whether MPT0B098 can block IL-6 induced JAK2/STAT3 signaling, we treated OEC-M1 and HSC-3 cells with IL-6 in the presence or absence of MPT0B098. The results showed that the phosphorylation of JAK2 and STAT3 was significantly increased by IL-6, and this induction was suppressed by MPT0B098 (Fig 6C). Moreover, MPT0B098 significantly induced apoptosis in OEC-M1 and HSC-3 cells, even in the presence of IL-6 (Fig 6D). These results clearly demonstrated that MPT0B098, by itself or in combination with other cancer drugs, can be useful therapy for OSCC.

## Discussion

Microtubule inhibitors, including microtubule stabilizers and destabilizers, are among the most active drugs used in cancer treatment. In this study, we identified a novel microtubule destabilizer, MPT0B098, which causes SOCS3 protein accumulation and consequently inhibits JAK2 and TYK2 activity, resulting in a loss of STAT3 phosphorylation and function. The loss of STAT3 activity sensitizes OSCC cells to MPT0B098-induced apoptosis. The mechanism by which MPT0B098 causes SOCS3 protein accumulation remains to be clarified. Because MPT0B098 treatment didn’t affect the mRNA level of SOCS3, it should be affecting the post-translational level of SOCS3. Pretreating OSCC cells with cycloheximide, a protein synthesis inhibitor, in the presence of MPT0B098 significantly extends the half-life of SOCS3 protein. Accumulated SOCS3 protein raises the probability that binding to its targets, such as JAK2 and TYK2, and facilitates the ubiquitination of JAK2 and TYK2 for further degradation. These findings indicate that the intact microtubule network is important for SOCS3 degradation. Disruption of microtubule network by MPT0B098 could interrupt the SOCS3 degradation and consequently prolongs the protein turnover rate. Similar results were observed in another microtubule-deploymerizing drug, nocodazole, which also affects the stability of SOCS1 proteins [26, 29]. Vuong et al. suggest that nocodazole disturbs the microtubule cytoskeleton and inhibits the SOCS1 degradation through the microtubule organizing complex-associated 20S proteasome pathway. Whereas Zou et al. show that inhibition of STAT3 phosphorylation by SOCS3 is reduced in nocodazole/IL-6 treated macrophages [30]. These findings point out a complex role of microtubule targeting agents for the negative regulating mechanism of SOCS3 in JAK2/STAT3 signaling. However, the precise molecular mechanisms of MPT0B098 in modulating SOCS3 stability must be further performed to elucidate.

Our results also indicate that MPT0B098-induced apoptosis ability was inverse correlation with endogenous STAT3 protein level. TW2.6 and SCC-15 which express high level of STAT3 protein are less sensitive to the cytotoxic effects of MPT0B098 than other OSCC cells which express low level of STAT3 protein. Inhibition of STAT3 leads to a sensitization of OSCC cells to MPT0B098 cytotoxicity, indicating that STAT3 is a key mediator of drug resistance and anti-apoptosis in oral carcinogenesis. Thus, in addition to its role in disrupting microtubule structure, microtubule-targeted agent MPT0B098 also suppress STAT3 signaling. Recently, the role of STAT3 has attracted significant attention as constitutive activation has been demonstrated in HNSCC, including OSCC [4, 31, 32]. Constitutive activation of STAT3 contributes to metastasis and chemotherapy resistance [33]. As such, targeting STAT3 is a potentially feasible approach to the treatment of cancer and several inhibitors of STAT3 are currently under development [34–36]. However, our results presented here suggest that inhibition of STAT3 may be an important effect of the MPT0B098 on OSCC cells that contain overexpressed STAT3. In addition, 5-FU and cisplatin are Food and Drug Administration (FDA) approved combinational drugs and routinely used following surgery or radiotherapy in HNSCC [15, 37]. A combinatorial treatment may improve the efficacy of these chemotherapeutic drugs and reduce their non-specific toxicities [18]. Our results showed that the combination of MTP0B098 with 5-FU or cisplatin exhibited significant higher growth inhibition and enhanced apoptosis in oral cancer cells as compared to treatment with a single drug or in combination of 5-FU and cisplatin. Therefore, we herein report the promising therapeutic potential and provide a novel strategy of MPT0B098 with 5-FU or cisplatin combinations as a new treatment for OSCC.

In conclusion, we identified a unique microtubule inhibitor, MPT0B098, which displays a novel mechanism to inhibit JAK2/STAT3 activity through SOCS3. In addition, we also provide a possibility that the combination of MPT0B098 with STAT3 inhibitors, 5-FU or cisplatin may work in synergy to present a useful therapeutic strategy in OSCC.

## Supporting Information

S1 Fig MPT0B098 inhibits microtubules polymerization. OEC-M1 and HSC-3cells were incubated at 37°C from 30 min in the presence of MPT0B098. Free form and polymer form of microtubule were purified and assessed by Western blot analysis.
(PDF)

S2 Fig Effect of SOCS2 on JAK2/STAT3 pathway. OEC-M1 cells were tranfected with control vector (VC) and construct containing the SOCS2-coding region (SOCS2) for 48 hrs. The phosphorylated proteins (p-JAK2, p-TYK2, p-STAT3 and p-STAT5) and total level of proteins (SOCS3, JAK2, TYK2, p-STAT3 and p-STAT5) were determined by Western blotting. GAPDH was used as loading control.
(PDF)

## References

[1] Yu H, Jove R. The STATs of cancer—new molecular targets come of age. Nat Rev Cancer. 2004;4(2):97–105. 10.1038/nrc1275 . 14964307

[2] Bromberg J. Stat proteins and oncogenesis. J Clin Invest. 2002;109(9):1139–42. 10.1172/JCI15617 . 11994401 PMC150969

[3] Carpenter RL, Lo HW. STAT3 Target Genes Relevant to Human Cancers. Cancers. 2014;6(2):897–925. 10.3390/cancers6020897 . 24743777 PMC4074809

[4] Grandis JR, Drenning SD, Zeng Q, Watkins SC, Melhem MF, Endo S, et al Constitutive activation of Stat3 signaling abrogates apoptosis in squamous cell carcinogenesis in vivo. Proc Natl Acad Sci U S A. 2000;97(8):4227–32. . 10760290 10.1073/pnas.97.8.4227 PMC18206

[5] Macha MA, Matta A, Kaur J, Chauhan SS, Thakar A, Shukla NK, et al Prognostic significance of nuclear pSTAT3 in oral cancer. Head & neck. 2011;33(4):482–9. 10.1002/hed.21468 . 20652980

[6] Grandis JR, Drenning SD, Chakraborty A, Zhou MY, Zeng Q, Pitt AS, et al Requirement of Stat3 but not Stat1 activation for epidermal growth factor receptor- mediated cell growth In vitro. J Clin Invest. 1998;102(7):1385–92. 10.1172/JCI3785 . 9769331 PMC508986

[7] Masuda M, Wakasaki T, Suzui M, Toh S, Joe AK, Weinstein IB. Stat3 orchestrates tumor development and progression: the Achilles' heel of head and neck cancers? Current cancer drug targets. 2010;10(1):117–26. . 20088788 10.2174/156800910790980197

[8] Siavash H, Nikitakis NG, Sauk JJ. Signal transducers and activators of transcription: insights into the molecular basis of oral cancer. Critical reviews in oral biology and medicine: an official publication of the American Association of Oral Biologists. 2004;15(5):298–307. . 15470267 10.1177/154411130401500505

[9] Tamiya T, Kashiwagi I, Takahashi R, Yasukawa H, Yoshimura A. Suppressors of cytokine signaling (SOCS) proteins and JAK/STAT pathways: regulation of T-cell inflammation by SOCS1 and SOCS3. Arteriosclerosis, thrombosis, and vascular biology. 2011;31(5):980–5. 10.1161/ATVBAHA.110.207464 . 21508344

[10] Croker BA, Krebs DL, Zhang JG, Wormald S, Willson TA, Stanley EG, et al SOCS3 negatively regulates IL-6 signaling in vivo. Nat Immunol. 2003;4(6):540–5. 10.1038/ni931 . 12754505

[11] Yasukawa H, Ohishi M, Mori H, Murakami M, Chinen T, Aki D, et al IL-6 induces an anti-inflammatory response in the absence of SOCS3 in macrophages. Nat Immunol. 2003;4(6):551–6. 10.1038/ni938 . 12754507

[12] Weber A, Hengge UR, Bardenheuer W, Tischoff I, Sommerer F, Markwarth A, et al SOCS-3 is frequently methylated in head and neck squamous cell carcinoma and its precursor lesions and causes growth inhibition. Oncogene. 2005;24(44):6699–708. 10.1038/sj.onc.1208818 . 16007169

[13] Warnakulasuriya S. Global epidemiology of oral and oropharyngeal cancer. Oral oncology. 2009;45(4–5):309–16. 10.1016/j.oraloncology.2008.06.002 . 18804401

[14] Jerjes W, Upile T, Petrie A, Riskalla A, Hamdoon Z, Vourvachis M, et al Clinicopathological parameters, recurrence, locoregional and distant metastasis in 115 T1-T2 oral squamous cell carcinoma patients. Head & neck oncology. 2010;2:9 10.1186/1758-3284-2-9 ; Central PMCID: PMC2882907. 20406474 PMC2882907

[15] Keil F, Selzer E, Berghold A, Reinisch S, Kapp KS, De Vries A, et al Induction chemotherapy with docetaxel, cisplatin and 5-fluorouracil followed by radiotherapy with cetuximab for locally advanced squamous cell carcinoma of the head and neck. European journal of cancer. 2013;49(2):352–9. 10.1016/j.ejca.2012.08.004 . 22981499

[16] Zhang N, Yin Y, Xu SJ, Chen WS. 5-Fluorouracil: mechanisms of resistance and reversal strategies. Molecules. 2008;13(8):1551–69. . 18794772 10.3390/molecules13081551 PMC6244944

[17] Fang L, Wang H, Zhou L, Yu D. FOXO3a reactivation mediates the synergistic cytotoxic effects of rapamycin and cisplatin in oral squamous cell carcinoma cells. Toxicology and applied pharmacology. 2011;251(1):8–15. 10.1016/j.taap.2010.11.007 . 21092744

[18] Sivanantham B, Sethuraman S, Krishnan UM. Combinatorial Effects of Curcumin with an Anti-Neoplastic Agent on Head and Neck Squamous Cell Carcinoma Through the Regulation of EGFR-ERK1/2 and Apoptotic Signaling Pathways. ACS combinatorial science. 2016;18(1):22–35. 10.1021/acscombsci.5b00043 . 26505786

[19] Wilken R, Veena MS, Wang MB, Srivatsan ES. Curcumin: A review of anti-cancer properties and therapeutic activity in head and neck squamous cell carcinoma. Molecular cancer. 2011;10:12 10.1186/1476-4598-10-12 . 21299897 PMC3055228

[20] Cheng YC, Liou JP, Kuo CC, Lai WY, Shih KH, Chang CY, et al MPT0B098, a novel microtubule inhibitor that destabilizes the hypoxia-inducible factor-1alpha mRNA through decreasing nuclear-cytoplasmic translocation of RNA-binding protein HuR. Mol Cancer Ther. 2013;12(7):1202–12. 10.1158/1535-7163.MCT-12-0778 . 23619299

[21] Shiah SG, Hsiao JR, Chang WM, Chen YW, Jin YT, Wong TY, et al Downregulated miR329 and miR410 promote the proliferation and invasion of oral squamous cell carcinoma by targeting Wnt-7b. Cancer Res. 2014;74(24):7560–72. 10.1158/0008-5472.CAN-14-0978 . 25351956

[22] Chang JY, Lai MJ, Chang YT, Lee HY, Cheng YC, Kuo CC, et al Synthesis and biological evaluation of 7-arylindoline-1-benzenesulfonamides as a novel class of potent anticancer agents. Medchemcomm. 2010;1(2):152–5. 10.1039/c0md00052c .

[23] Gundersen GG, Khawaja S, Bulinski JC. Postpolymerization detyrosination of alpha-tubulin: a mechanism for subcellular differentiation of microtubules. J Cell Biol. 1987;105(1):251–64. . 2886509 10.1083/jcb.105.1.251 PMC2114889

[24] Alexander WS. Suppressors of cytokine signalling (SOCS) in the immune system. Nat Rev Immunol. 2002;2(6):410–6. Epub 2002/07/03. 10.1038/nri818 . 12093007

[25] Shuai K, Liu B. Regulation of JAK-STAT signalling in the immune system. Nat Rev Immunol. 2003;3(11):900–11. Epub 2003/12/12. 10.1038/nri1226 . 14668806

[26] Vuong BQ, Arenzana TL, Showalter BM, Losman J, Chen XP, Mostecki J, et al SOCS-1 localizes to the microtubule organizing complex-associated 20S proteasome. Mol Cell Biol. 2004;24(20):9092–101. Epub 2004/10/01. 10.1128/MCB.24.20.9092-9101.2004 . 15456882 PMC517868

[27] Lesina M, Kurkowski MU, Ludes K, Rose-John S, Treiber M, Kloppel G, et al Stat3/Socs3 activation by IL-6 transsignaling promotes progression of pancreatic intraepithelial neoplasia and development of pancreatic cancer. Cancer cell. 2011;19(4):456–69. 10.1016/j.ccr.2011.03.009 . 21481788

[28] Sansone P, Bromberg J. Targeting the interleukin-6/Jak/stat pathway in human malignancies. Journal of clinical oncology: official journal of the American Society of Clinical Oncology. 2012;30(9):1005–14. 10.1200/JCO.2010.31.8907 . 22355058 PMC3341105

[29] Nishi M, Ryo A, Tsurutani N, Ohba K, Sawasaki T, Morishita R, et al Requirement for microtubule integrity in the SOCS1-mediated intracellular dynamics of HIV-1 Gag. FEBS Lett. 2009;583(8):1243–50. 10.1016/j.febslet.2009.03.041 . 19327355

[30] Zou T, Ouyang L, Chen L, Dong W, Qiao H, Liu Y, et al The role of microtubule-associated protein 1S in SOCS3 regulation of IL-6 signaling. FEBS Lett. 2008;582(29):4015–22. Epub 2008/11/26. 10.1016/j.febslet.2008.10.055 . 19027008

[31] Brown ME, Bear MD, Rosol TJ, Premanandan C, Kisseberth WC, London CA. Characterization of STAT3 expression, signaling and inhibition in feline oral squamous cell carcinoma. BMC veterinary research. 2015;11:206 10.1186/s12917-015-0505-7 . 26272737 PMC4536595

[32] Gkouveris I, Nikitakis N, Karanikou M, Rassidakis G, Sklavounou A. Erk1/2 activation and modulation of STAT3 signaling in oral cancer. Oncol Rep. 2014;32(5):2175–82. 10.3892/or.2014.3440 . 25174327

[33] Bu LL, Zhao ZL, Liu JF, Ma SR, Huang CF, Liu B, et al STAT3 blockade enhances the efficacy of conventional chemotherapeutic agents by eradicating head neck stemloid cancer cell. Oncotarget. 2015;6(39):41944–58. 10.18632/oncotarget.5986 . 26556875 PMC4747200

[34] Bendell JC, Hong DS, Burris HA 3rd, Naing A, Jones SF, Falchook G, et al Phase 1, open-label, dose-escalation, and pharmacokinetic study of STAT3 inhibitor OPB-31121 in subjects with advanced solid tumors. Cancer Chemother Pharmacol. 2014;74(1):125–30. 10.1007/s00280-014-2480-2 . 24819685

[35] Nelson EA, Walker SR, Kepich A, Gashin LB, Hideshima T, Ikeda H, et al Nifuroxazide inhibits survival of multiple myeloma cells by directly inhibiting STAT3. Blood. 2008;112(13):5095–102. 10.1182/blood-2007-12-129718 . 18824601 PMC2597607

[36] Sen M, Thomas SM, Kim S, Yeh JI, Ferris RL, Johnson JT, et al First-in-human trial of a STAT3 decoy oligonucleotide in head and neck tumors: implications for cancer therapy. Cancer discovery. 2012;2(8):694–705. 10.1158/2159-8290.CD-12-0191 . 22719020 PMC3668699

[37] Lorch JH, Goloubeva O, Haddad RI, Cullen K, Sarlis N, Tishler R, et al Induction chemotherapy with cisplatin and fluorouracil alone or in combination with docetaxel in locally advanced squamous-cell cancer of the head and neck: long-term results of the TAX 324 randomised phase 3 trial. The Lancet Oncology. 2011;12(2):153–9. 10.1016/S1470-2045(10)70279-5 . 21233014 PMC4356902

