# Supplementary figures and images for: MPT0B098, a Microtubule Inhibitor, Suppresses JAK2/STAT3 Signaling Pathway through Modulation of SOCS3 Stability in Oral Squamous Cell Carcinoma
Source: PLoS One. 2016 Jul 1;11(7):e0158440. doi: 10.1371/journal.pone.0158440 (PMC4930189; doi:10.1371/journal.pone.0158440)

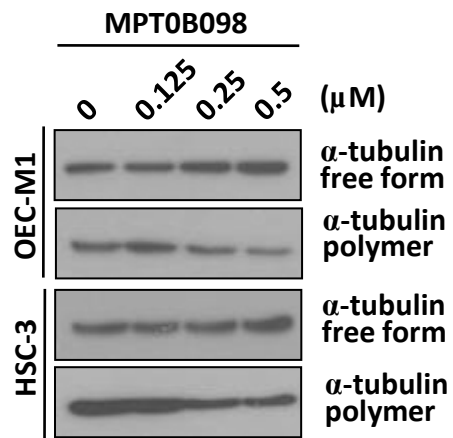

Supplement: S1 Fig — OEC-M1 and HSC-3cells were incubated at 37°C from 30 min in the presence of MPT0B098. Free form and polymer form of microtubule were purified and assessed by Western blot analysis. (PDF) [file pone.0158440.s001.pdf]

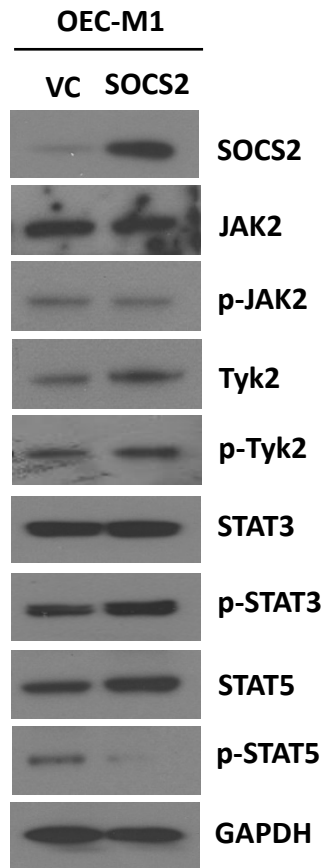

Supplement: S2 Fig — OEC-M1 cells were tranfected with control vector (VC) and construct containing the SOCS2-coding region (SOCS2) for 48 hrs. The phosphorylated proteins (p-JAK2, p-TYK2, p-STAT3 and p-STAT5) and total level of proteins (SOCS3, JAK2, TYK2, p-STAT3 and p-STAT5) were determined by Western blotting. GAPDH was used as loading control. (PDF) [file pone.0158440.s002.pdf]
